# Swallowing Evaluation in Post-COVID-19 Patients with Oropharyngeal Dysphagia

**DOI:** 10.1007/s00455-025-10810-w

**Published:** 2025-03-05

**Authors:** Ahmed Mohamed Zayed, Omayma Afsah, Tamer Elhadidy, Tamer Abou-Elsaad

**Affiliations:** 1 Phoniatric Unit, ORL Department, Faculty of Medicine, Mansoura University, Mansoura, 35516 Egypt; 2 Chest Diseases Department, Faculty of Medicine, Mansoura University, Mansoura, Egypt

**Keywords:** Post.COVID-19, Oropharyngeal Dysphagia, FEES

## Abstract

Oropharyngeal dysphagia (OD) is a prevalent issue in hospitalized COVID-19 patients. This study aimed to determine swallowing abnormalities in post-COVID-19 patients with OD and to determine the potential risk factors of aspiration in patients who have recovered from COVID-19. Screening for OD was done for 310 patients who were discharged from the main university isolation hospital during the study period. A longitudinal descriptive study was carried out on 127 adult post-COVID-19 patients between the ages of 24 and 65 years who failed OD screening at the time of discharge. Instrumental swallowing assessment was done using fiberoptic endoscopic evaluation of swallowing (FEES) at one of two different time points: one-week post-discharge (Group 1) and 3–4 weeks post-discharge (group 2). The prominent swallowing abnormalities were delayed triggering of swallowing reflex, laryngeal penetration, tracheal aspiration, as well as vallecular and pyriform sinuses residue with lower frequencies and milder degrees in group 2 than in group 1 patients. Statistically significant associations were found between the presence of ageusia and anosmia in post-COVID-19 patients and both impaired laryngeal sensation and delayed triggering of the swallowing reflex. Significant associations were detected between aspiration in post-COVID-19 patients and the following factors: higher Eating Assessment Tool (EAT-10) scores, presence of dysphonia, higher respiratory rate, and the longer duration of the use of noninvasive ventilation (NIV) and/or invasive mechanical ventilation (IMV). The combined higher EAT-10 scores and higher respiratory rate predicted aspiration in post-COVID-19 patients by an overall percentage of 87.1%

## Introduction

The coronavirus disease 2019 (COVID-19) pandemic has spread worldwide. It is due to the severe acute respiratory syndrome coronavirus 2 (SARS-CoV-2) [1]. Patients convalescing from severe or critical COVID-19 present several impairments, including dysphagia, respiratory sequelae, cognitive changes, nervous system disorders, myopathy, and neuropathy [2]. In COVID-19 patients, oropharyngeal dysphagia (OD) is a serious concern. It is a prevalent issue among patients discharged from the intensive care unit (ICU), particularly those undergoing invasive mechanical ventilation (IMV), having tracheostomies, having severe chest infections, respiratory insufficiency, or pneumonia [3], and is strongly related to critical care outcomes [4]. Oropharyngeal dysphagia is also a possibility for COVID-19 patients who are admitted to the ward during the viral response phase, pulmonary phase, or hyper-inflammation phase [5]. According to estimates, 35% of hospitalized COVID-19 patients had OD, which is linked to a higher risk of mortality [6]. Holdiman et al. [7] suggested that pneumonia, acute respiratory distress syndrome (ARDS), prone positioning, ventilation, and intubation are significant predictors of OD development in patients hospitalized with COVID-19.

COVID-19 patients, especially those with moderate-to-severe disease, experienced OD not only during but also after recovery from the disease [8]. However, specific information related to the etiology and nature of post-COVID-19 dysphagia has not been confirmed [9]. OD in post-acute COVID-19 infection probably derives from a diversity of supposed causes. The most widely accepted explanation for dysphagia following COVID-19 is that the cranial nerves in charge of the swallowing reflex are involved [10]. Frank et al. [11] contended that the fundamental cause of OD in post-COVID-19 patients is intrinsic impairment of the central and peripheral neuronal swallowing network. Prolonged intubation may also impede airway sensations, which could decrease the protective cough response even when aspiration occurs [10]. Continuous positive airway pressure (CPAP) or high-flow nasal oxygen (HFNO) can cause hypoesthesia, delayed swallow reflex triggering, breathing swallowing incoordination, and weak cough reflex by negatively affecting the pharyngeal and laryngeal chemo- and mechanoreceptors [12]. Marchese et al. [13] suggested that OD in post-COVID-19 patients may have a psychogenic etiology.

The EAT-10 is recommended as a simple and quick screening measure for OD. "The EAT-10 may be utilized as a clinical instrument to document the initial dysphagia severity and monitor the treatment response in persons with a wide array of swallowing disorders" [14]. The evidence-based "Yale swallow protocol" is a widely used screening method that can determine the risk of aspiration and, if passed, can suggest particular oral diets without requiring additional instrumental dysphagia testing [15].

Due to their increased risk of respiratory issues, the swallowing efficiency and safety of COVID-19 survivors must be evaluated carefully [16]. Lagier et al. [17] described swallowing findings only at the early stages of recovery in post-extubation COVID-19 patients using a videofluoroscopic swallowing study (VFSS). A high prevalence of aspiration and a lack of protective reflexes were reported. The primary aim of this study was to determine swallowing abnormalities in post-COVID-19 patients with oropharyngeal dysphagia (OD). The secondary aim was to determine the potential aspiration risk factors in post-COVID-19 patients.

## Subjects and Methods

### Subjects

A descriptive longitudinal study was carried out on 127 adult post-COVID-19 patients who failed OD screening at discharge from the main university isolation hospital from July 2021 to March 2022. According to the isolation hospital policy, all patients were admitted with severe or critical illness. Severe illness was defined as individuals who had an oxygen saturation measured by pulse oximetry (SpO_2_) ≤ 94% on room air, a ratio of partial pressure of arterial oxygen to fraction of inspired oxygen (PaO2/FiO2) < 300 mmHg, marked tachypnea with respiratory frequency > 30 breaths/min, or lung infiltrates > 50%. Critical illness was defined as individuals who had acute respiratory failure, septic shock, and/or multiple organ dysfunctions [18]. Patients with severe illness received either conventional oxygen therapy (COT) or noninvasive ventilation (NIV), while patients with critical illness received either NIV or invasive mechanical ventilation (IMV). Patients under the age of eighteen and older than sixty-five, stroke patients, patients with previous OD, patients who have had OD because of neurological diseases or head and neck malignancies, and those with tracheostomies were excluded from the study. All participants provided written informed consent before the study.

### Methods

All participants underwent the following assessment protocol at the time of discharge from the hospital:

(A) Patient history:

- Demographic information (name, age, and gender).
- Medical history: including history of previous OD, neurological disorders such as stroke, or head and neck cancer. COVID-19 infection was established in the admitted patients by detecting SARS-CoV-2 RNA employing reverse transcription-polymerase chain reaction (RT-PCR). Patients with respiratory clinical syndrome consistent with acute infection were included in the study.
- Present history: including length of hospital stay, ageusia, anosmia, and dysphonia, as well as duration and method of delivery of oxygen therapy (Conventional oxygen therapy, non-invasive ventilation (NIV), and IMV).

(B) Clinical examination: this involves evaluating the patient's respiratory status by measuring heart rate, respiratory rate, and oxygen saturation using pulse oximetry.

(C) Screening for OD with the Arabic version of the "Eating Assessment Tool" (EAT-10)” [19] and the evidence-based "Yale swallow protocol" [15].

**-** The Arabic version of the "Eating Assessment Tool" (EAT-10)” is a self-administered questionnaire designed to assess OD in the Arabic-speaking population in a subjective manner. There are ten statements total, each given a 5-point rating on a scale from 0 (indicates no problem) to 4 (indicates severe problem). A summated EAT-10 total score falls between 0 and 40; a score of ≥ 3 indicates dysphagia. One's self-perception of dysphagia is higher when their EAT-10 score is elevated.

- The "Yale swallow protocol" (bedside swallowing assessment) involved three items:

I. Brief cognitive screen.

II. Oral mechanism examination.

III. The patient had a 3-oz water swallow test, the results of which were evaluated based on the following standards:

"PASS": Drinking 90 ml (3 oz) of water entirely and continuously without exhibiting overt aspiration signs, such as coughing or choking, during or right after the task.

"FAIL": The patient stopped/started swallowing before finishing the whole 3 oz or showed overt aspiration signs, such as coughing or choking, during or right after the task.

**Fig. 1:**
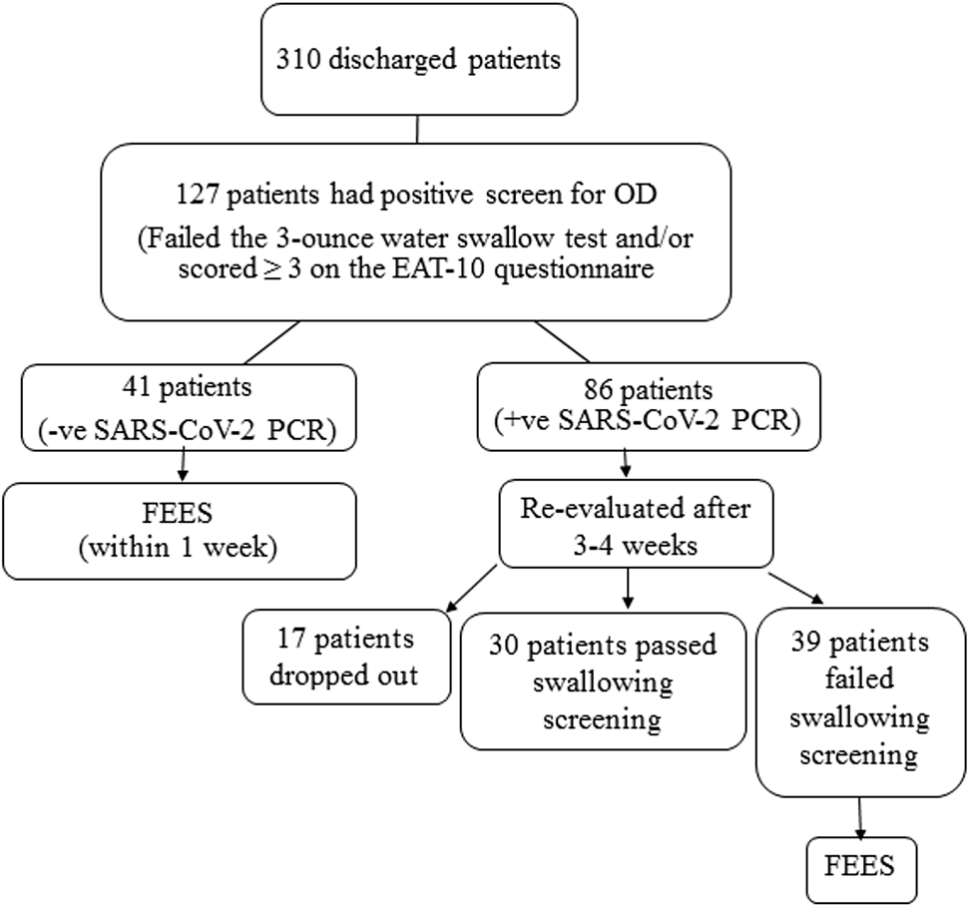
Flowchart of patient enrollment/exclusions and assessments

Adequate precautions were taken during the bedside swallowing assessment to avoid transmission of infection to the clinician. The phoniatrician was positioned at the patient's side, maintaining a distance of about 2 m [20]. Patients who had a positive screen for OD (failed the 3-oz water swallow test and/or scored ≥ 3 on the EAT-10 questionnaire) were included in the study. These were 127 patients out of 310 discharged patients who were screened for OD during the study period. The timing of fiberoptic endoscopic evaluation of swallowing (FEES) was determined according to the result of SARS-CoV-2 PCR at the time of discharge based on infection control measures and the potential persistence of viral RNA. Patients with negative SARS-CoV-2 PCR were evaluated by FEES within one week after discharge (Group 1 patients). For patients who had positive SARS-CoV-2 PCR, FEES was postponed, and compensatory treatment strategies (including bolus modification and postural techniques) or nasogastric tube feeding were considered till the 1st follow-up visit at 3–4 weeks after discharge when patients were reevaluated by screening tools at post-COVID-19 clinic in the university hospital. Patients who still failed OD screening were evaluated by FEES (Group 2 patients). Counting from the onset of COVID-19 symptoms, group 1 patients were considered in the early stage of recovery, and group 2 patients were considered in the late stage of recovery. The number of patients who underwent FEES was 80 (41 in group 1 and 39 in group 2) (Fig. 1).

(D) Instrumental swallowing assessment:

- It was performed through fiberoptic endoscopic evaluation of swallowing (FEES). FEES was done using fiberoptic nasendoscopy “Henke-Sass-Wolf, type10” linked to a Lemke video camera (MC204) and software for recording and retrieving recorded materials. Personal protective equipment was used during the FEES.

- FEES Examination protocol [21] was used:

Part 1: Anatomic- physiologic assessment:

A. Visualization of pharynx and larynx at rest:

B. Secretions and how they were handled:

- The quantity, location, and reaction of the patient to secretions were noted over 2 min.
- The Murray secretion scale (MSS) [22], a four-grade scale, was used to rate the pooling of secretions in the hypopharynx and larynx before the first swallow. A patient was given score "1" when no visible or just a few bubbles of secretion were observed in the vallecula and hypopharynx, score "2" for deeply pooled secretions in the vallecula and pyriform sinus, score "4" when secretions were found in the laryngeal vestibule, and score "3" for any secretion that altered from a “2” to a “4” rating during the examination.
**-** Ice chip protocol was considered if secretions were observed in the laryngeal vestibule and the patient could not swallow saliva successfully.

C. Laryngeal function during both respiration and phonation.

D. Sensory testing:

- Laryngeal sensation was examined using the touch method, which involved touching the arytenoid cartilages by the tip of the flexible laryngoscope. The laryngeal sensation was considered to be grossly intact if any of the following signs were observed: laryngeal adductor reflex (LAR), a cough, a gag, eye blinking, tearing, throat clearing, swallowing, or a patient’s report of feeling the touch [23].

Part 2: Swallowing food and liquid:

During FEES, boluses with varying volumes and consistencies tinted green using a coloring agent were used: fluids (3 ml, 5 ml, 10 ml, and cup drinking), puree food (10 ml yogurt), and solid food (half biscuit) with two trials for each consistency. The patient's clinical status and swallowing function were considered when selecting the appropriate boluses. If aspiration occurred twice with any bolus amount or consistency and compensatory strategies failed to eliminate aspiration, these boluses were deemed unsafe for the patient.

- Swallowing function during FEES was assessed using two standard rating scales from video recordings: The penetration-aspiration scale (PAS) [24] and the Mansoura fiberoptic endoscopic evaluation of swallowing residue rating scale (MFRRS) [25]. The PAS is an eight-point rating scale that considers the material's depth into the airway and its clearance from the airway during swallowing trials. MFRRS is an accurate, anatomically based tool that is highly reliable for judging post-swallow pharyngeal residue during FEES. Two 7-point ordinal scales are used in MFRRS (one for pyriform sinus residue and one for vallecular residue).

Part 3: Therapeutic interventions:

Compensatory interventions were combined with "part 2" and trialed as soon as applicable. These included postural techniques, bolus modifications, and behavioral adjustments (e.g., washing down residue with liquid).

### Statistical analysis

IBM SPSS software, version 22.0, was used to input and analyze data. Numbers and percentages were used to describe the qualitative data. Quantitative data were characterized using the median (minimum and maximum) for non-parametric data and the mean and standard deviation for parametric data. Acquired results were deemed significant at the (0.05) level. The Chi-Square test was employed to compare two or more groups. For parametric tests, two independent groups were compared using the student t-test; for non-parametric tests, two independent groups were compared using the Mann–Whitney U test. The study employed stepwise logistic regression analysis to forecast independent variables associated with a binary outcome. In the univariate analysis, significant predictors were entered.

## Results

1- Descriptive data of the entire group:

This study was carried out on 127 patients, including 81 females (63.8%) and 46 males (36.2%) in the age range of 24–65 years (mean 55.2 ± 9.43). At the time of hospital discharge, 42.5% of the patients had dysphonia, and 15.7% had aguesia and anosmia. The range of hospital stay of patients was 4—42 days (median = 8 days). Conventional oxygen therapy was delivered for 34 patients, NIV for 86 patients, and only seven received IMV (Table 1). The type of respiratory support received by COVID-19 severity categories is presented in Table 2.

**Table 1:** Demographic and basic data of the entire group (N = 127)

	Mean ± SD/Median (Range)
Age (Years)	
Mean ± SD	55.2 ± 9.34
(Range)	(24–65)
Oxygen saturation	
Mean ± SD	95.34 ± 2.56
(Range)	(86–99)
Respiratory rate	
Mean ± SD	20.5 ± 2.21
(Range)	(14–24)
Duration of hospital stay (in days)	
Median (Range)	8.0 (4–42)
	Number (%)
Gender	
Male	46 (36.2%)
Female	81 (63.8%)
Aguesia and anosmia	20 (15.7%)
Dysphonia	54 (42.5%)
Oxygen therapy	
Conventional oxygen therapy	34 (26.8%)
Non-invasive ventilation (NIV)	86 (67.7%)
Invasive mechanical ventilation (IMV)	7 (5.5%)

**Table 2:** Type of respiratory support received by COVID-19 severity categories (N = 127)

COVID-19 severity category	Respiratory support		
	Conventional oxygen therapy (COT)	Noninvasive ventilation (NIV)	Invasive mechanical ventilation (IMV)
Severe illness	34 (27%)	58 (46%)	–
Critical illness	–	28 (22%)	7 (5%)

2- Results of instrumental swallowing assessment of patients:

a) Group 1:

Out of the 127 discharged patients in our study who screened positive for oropharyngeal dysphagia, 41 patients (32.3%) were discharged with negative SARS-CoV-2 PCR. The mean hospital stay duration in this group was 17.2 ± 9.5 days. Thirty-seven out of these 41 patients (90.2%) were admitted to the ICU (mean duration of ICU stay was 14.4 ± 7.3 days). This group included the seven patients who received IMV. Patients were evaluated by FEES within one week after the date of discharge. At the time of FEES, 26 patients (63.4%) received their nutrition via a nasogastric tube (NGT), and 14 continued feeding through an NGT following the FEES examination. Pooling of secretions before the introduction of the first bolus was found in 15 patients (36.6%), with 26.7% (4 out of 15) having consistent secretions in the laryngeal vestibule (grade "4" on the Murray rating scale). Tracheal aspiration was evident in 25 patients (61%), with 20% (5 out of 25) being silent aspirators (grade 8 on PAS). Aspiration mainly occurred on fluid boluses. Vallecular and hypopharyngeal residue was a salient finding in a majority of patients. It mainly occurred after the intake of puree and solid food boluses and was more common in the vallecula than in the pyriform sinuses. Thirty-five patients (85.4%) had residue in the vallecula, scoring grade 2 or more, according to MFRRS. In comparison, 24 patients (58.5%) had residue in the pyriform sinuses scoring grade 2 or more according to MFRRS (Table 3).

**Table 3:** FEES findings in post-COVID-19 patients (n = 80)

	Group 1 (G1) N = 41	Group 2 (G2) N = 39
(A) Anatomic-physiologic findings		
Number (%)		
Murray secretion scale (MSS)		
* Score 1	26(63.4)	39(100)
* Score 2	6(14.6)	0(0)
* Score 3	5(12.2)	0(0)
* Score 4	4(9.8)	0(0)
VF immobility (Unilateral)	9(22)	6(15.4)
Laryngeal sensation		
Intact	25(61)	38(97.4)
Impaired	16(39)	1(2.6)
(B) Swallowing findings		
Number (%)		
Delayed triggering of the swallowing reflex	25(61.0)	5(12.8)
Penetration-Aspiration Scale (PAS)		
* Score 1	1(2.4)	26(66.7)
* Score 2	4(9.8)	2(5.1)
* Score 3	2(4.9)	2(5.1)
* Score 4	8(19.5)	1(2.6)
* Score 5	1(2.4)	2(5.1)
* Score 6	11(26.8)	6(15.4)
* Score 7	9(22.0)	0(0)
* Score 8	5(12.2)	0(0)
Penetration	15(36.6)	7(17.9)
Aspiration	25(61.0)	6(15.4)
Vallecular residue scale (MFRRS)		
* Score 0	6(14.6)	6(15.4)
* Score 1	0(0)	15(38.5)
* Score 2	10(24.4)	16(41.0)
* Score 3	10(24.4)	2(5.1)
* Score 4	6(14.6)	0(0)
* Score 5	6(14.6)	0(0)
* Score 6	3(7.3)	0(0)
Pyriform sinus residue scale (MFRRS)		
* Score 0	7(17.1)	18(46.2)
* Score 1	10(24.4)	18(46.2)
* Score 2	13(31.7)	3(7.7)
* Score 3	3(7.3)	0(0)
* Score 4	4(9.8)	0(0)
* Score 5	2(4.9)	0(0)
* Score 6	2(4.9)	0(0)

b) Group 2:

Out of the 127 discharged patients, 86 (67.7%) were discharged with positive SARS-CoV-2 PCR. Out of these 86 patients, 17 dropped out, and 69 continued the study. All patients in this group received COT or NIV. Their mean hospital stay was 15.1 ± 5.9 days. They were re-evaluated by swallowing screening tools at a post-COVID-19 clinic in the university hospital 3–4 weeks after discharge. Thirty (43.5%) patients passed swallowing screening tools (EAT-10 total score < 3 and passed Yale swallow protocol) with no further assessment done. FEES was done for 56.5% of patients (39 out of 69) (EAT-10 total score ≥ 3 or failed Yale swallow protocol), with the mean hospital stay duration for these 39 patients being 14.8 ± 7 days. 64.1% (25 out of 39) were admitted to the ICU with a mean duration of (6.84 ± 5.3 days). Tracheal aspiration was evident in only 6 patients (15.4%) with no silent aspirators. 16 patients (41%) had vallecular residue post swallowing “grade 2 (Shallow pooling),” and only 2 patients had “grade 3 (Moderate pooling),” according to MFRRS. Residue in the pyriform sinuses was found in only 3 patients who had grade 2 (Shallow pooling), according to MFRRS (Table 3).

**3**- Comparative analysis of several assessment parameters in post-COVID-19 patients with aspiration and patients without aspiration as determined by FEES:

Compared to patients without aspiration, patients with aspiration had a significantly higher respiratory rate, required noninvasive and/or invasive mechanical ventilation for a more extended period, and had higher EAT-10 scores. Additionally, the percentage of patients with dysphonia was considerably higher in the aspiration group. 32.7% of patients who received NIV (18 out of 55) had aspiration, while 85.7% of patients who received IMV (6 out of 7) had aspiration (Table 4).

**Table 4:** Comparison between patients with aspiration and patients without aspiration

	Patients without aspiration n = 49	Patients with aspiration n = 31	Test of significance
Age (Years)			t = 1.68
Mean ± SD	57.61 ± 8.83	54.09 ± 9.52	P = 0.096
Gender			
Male	18(36.7%)	14 (45.2%)	χ^2^ = 0.562
Female	31(63.3%)	17 (54.8%)	P = 0.454
Duration of hospital stay (in days)			z = 0.470
Median (Range)	13(4–34)	14(6–42)	P = 0.638
Aguesia and anosmia			
Absent	40(81.6%)	20 (64.5%)	χ^2^ = 2.97
Present	9(18.4%)	11 (35.5%)	P = 0.085
Dysphonia			
Absent	24(49.0%)	2 (6.5%)	χ^2^= 15.65
Present	25(51.0%)	29 (93.5%)	P < 0.001*
Respiratory rate			t = 2.53
Mean ± SD	20.86 ± 3.03	22.58 ± 2.88	P = 0.014*
EAT-10 score	5(3–12)	15(3–18)	z = 4.81
Median (Range)			P < 0.001*
NIV/IMV			
No	11(22.4%)	7 (22.6%)	χ^2^= 7.35
NIV	37(75.5%)	18 (58.1%)	P = 0.025*
IMV	1(2.0%)	6 (19.4%)	
Duration of NIV/IMV (Days)	6(2–21)	14(7–32)	z = 4.43
Median (Range)			P < 0.001*

Tests used: (t) = Student t-test, (χ2) = Chi-Square test, (z) = Mann–Whitney U test, *P < 0.05 = statistically significant, *NIV* Noninvasive ventilation, *IMV* Invasive mechanical ventilation

Data expressed as mean ± SD, Median (Range) or as frequency (Number-percent)

*SD* standard deviation

4- Association between the presence of ageusia and anosmia, laryngeal sensation, and triggering of swallowing reflex:

Significant associations were found between the presence of ageusia and anosmia, impaired laryngeal sensation, and delayed triggering of the swallowing reflex (Table 5).

**Table 5:** Relation between the presence of aguesia and anosmia, laryngeal sensation, and triggering of the swallowing reflex

	Aguesia and anosmia		Test of significance
	Absent N = 60	Present N = 20	
Laryngeal sensation:			
Intact	54 (90.0%)	9 (45%)	χ^2^ = 15.47
Impaired	6 (10.0%)	11(55%)	P < 0.001*
Triggering of swallowing reflex:			
Intact	46 (76.7%)	4 (20.0%)	χ^2^ = 20.55
Delayed	14 (23.3%)	16 (80.0%)	P < 0.001*

Test used: (χ2): Chi-Square test

^*^statistically significant

5- Risk factors of aspiration in post-COVID-19 patients:

Significant associations were observed between aspiration in post-COVID-19 patients and the following factors: higher EAT-10 scores, presence of dysphonia, higher respiratory rate, and the longer duration of the use of NIV and/or IMV. A higher EAT-10 score was associated with a 1.31 higher incidence of aspiration, and every 1-day increase in the duration of NIV and/or IMV use increased the risk of aspiration by 1.09. Multivariate analysis revealed that the combined higher EAT-10 scores and higher respiratory rate were considered significant risk factors, with an adjusted odds ratio of 1.31 and 0.57, respectively. The overall percentage of predicted aspiration by both factors was 87.1% (Table 6).

**Table 6:** Univariate and multivariate analyses of the aspiration risk factors

Variables	Total number N = 80	Aspiration		Univariate analysis		Multivariate analysis	
				p	Crude Odds ratio (95% CI)	p	Adjusted Odds ratio (95% CI)
		Absent (N = 49)	Present (N = 31)				
Age (Years)							
Mean ± SD	80	57.61 ± 8.83	54.09 ± 9.52	0.101	0.959(0.912–1.01)		
Gender							
Male	32	18(36.7%)	14 (45.2%)	0.454	1.42(0.568–3.54)		
Female( R)	48	31(63.3%)	17 (54.8%)				
Length of hospital stay (Days)							
Median (range)	80	13(4–34)	14(6–42)	0.179	1.04(0.983–1.09)		
Ageusia and anosmia							
Absent (R)	60	40(81.6%)	20 (64.5%)	0.089	2.44(0.871–6.86)		
Present	20	9(18.4%)	11 (35.5%)				
Dysphonia							
Absent (R)	26	24(49.0%)	2 (6.5%)	0.001*	13.92(2.99–64.83)	0.094	7.53(0.707–80.09)
Present	54	25(51.0%)	29 (93.5%)				
Respiratory rate							
Mean ± SD	80	20.86 ± 3.03	22.58 ± 2.88	0.017*	1.22(1.04–1.44)	0.028*	0.573(0.349–0.941
EAT-10 score							
Median (Range)	80	5(3–12)	15(3–18)	< 0.001*	1.42(1.22–1.66)	0.002*	1.31(1.25–1.9)
NIV/IMV							
No (R)	18	11(22.4%)	7 (22.6%)	0.079	1		
NIV	55	37(75.5%)	18 (58.1%)	0.633	0.764(0.254–2.30)		
IMV	7	1(2.0%)	6 (19.4%)	0.058	9.43(0.927–95.88)		
Duration of NIV/IMV (Days)							
Median (Range)	80	6(2–21)	14(7–32)	< 0.001*	1.24(1.11–1.39)	0.270	1.09(0.931–1.29)
	Overall % predicted = 87.1%						
	Modelχ^2^ = 42.69, p < 0.001*						

*R* reference group

^*^p < 0.05 = statistically significant, *NIV* Noninvasive ventilation, *IMV* Invasive mechanical ventilation

## Discussion

Recovery profiles for COVID-19 patients with OD and its prevalence are unknown, although some reports suggest it may be frequent [26]. Zayed et al. [27] reported that 45.4% of the admitted and 40.97% of the discharged COVID-19 patients had a positive screen for OD. In our study, instrumental swallowing assessment of post-COVID-19 patients using FEES revealed several abnormalities in groups 1 and 2. In group 1, delayed triggering of swallowing reflex was evident in 61% of patients, and impaired laryngeal sensation was found in 39%. Tracheal aspiration was evident in 61% of patients, with one-fifth being silent aspirators (grade 8 on PAS). Consistent with these findings, Lagier et al. [17] described the delayed pharyngeal phase as the most frequent finding among the early videofluoroscopic swallowing study (VFSS) findings in severe COVID-19 patients who recovered from acute respiratory distress syndrome (ARDS). Additionally, a high prevalence of aspiration and lack of protective reflexes were reported. The prevalence of silent aspiration in the Lagier et al. [17] study was 80%, most probably because all of their patients were post-extubated from mechanical ventilation. Vallecular and hypopharyngeal residue was another salient finding in most patients during the initial post-discharge evaluation. This could be due to reduced propulsion by the tongue base and impaired pharyngeal peristalsis. In group 2, delayed triggering of swallowing reflex, laryngeal penetration, tracheal aspiration, and vallecular and pyriform sinuses residue were noted but with lower frequencies and milder degrees than those noted in group 1. Llados et al. [28] suggested that vagus nerve dysfunction (VND) mediated by SARS-CoV-2 could elucidate some persistent COVID-19 symptoms, such as dysphonia and dysphagia.

In the current study, 43.5% of post-COVID-19 patients who were re-evaluated by swallowing screening tools at 3–4 weeks post-discharge passed OD screening and did not require instrumental swallowing assessment. Notably, these patients received either conventional oxygen therapy or NIV during their hospital stay. This finding could be due to the self-limiting nature of dysphagia among non-intubated patients with COVID-19, as reported by Grilli et al. [29], who mentioned that swallowing impairment in non-intubated COVID-19 patients showed a natural tendency to spontaneous resolution in the majority of cases. Additionally, prolonged dysphagia could be attributed to the use of IMV with its hazardous effect on swallowing function. As mentioned by Rassameehiran et al. [30], post-extubation dysphagia has multiple underlying causes, including mechanical factors, cognitive disturbances, and the long-term effects of narcotics and anxiolytic drugs. The length of intubation and the size of the endotracheal tube are directly related to mechanical causes because these tubes induce mucosal inflammation that results in loss of architecture, atrophy of the oropharyngeal muscles from intubation-related disuse, reduced proprioception, decreased laryngeal sensation, and laryngeal injury (edema, granuloma, and vocal fold paralysis).

Statistically significant associations were found between the presence of ageusia and anosmia in post-COVID-19 patients and both impaired laryngeal sensation and delayed triggering of the swallowing reflex. This could be explained by the fact that swallowing, a nutritional process that involves the oral preparatory phase, depends on the interplay of several senses, such as taste and smell. Taste, in particular, contributes to the perception of different consistencies, functionality of protective reflexes, and oropharyngeal motor response [31]. As reported by Descamps et al. [32], SARS-CoV-2 attacks human cells via the angiotensin-converting enzyme 2 (ACE2) receptors, which have a significant expression in the upper aerodigestive tract, including olfactory epithelium, oral mucosa, salivary glands, pharyngeal and laryngeal epithelia. Doblan et al. [33] found that the SARS-CoV-2 virus commonly causes cranial nerve dysfunction symptoms with more sensory dysfunction than motor involvement.

Tracheal aspiration is a significant contributing factor to aspiration pneumonia. In patients with COVID-19, aspiration can worsen their already compromised lung function [34]. Therefore, performing FEES for patients with COVID-19 and negative SARS-CoV-2 PCR results would be preferable as soon as feasible. In the present study, post-COVID-19 patients with aspiration showed a significantly higher respiratory rate, longer NIV and/or IMV use duration, and higher EAT-10 scores than patients without aspiration. Cvejic et al. [35] stated that an increase in the baseline respiratory rate is associated with swallowing dysfunction and a high risk of aspiration related to breathing–swallowing discoordination. Hori et al. [36] demonstrated that the occurrence rate of aspiration after the swallow was more significant with the use of bi-level positive airway pressure (BiPAP). Oomagari et al. [12] and Arizono et al. [37] highlighted the effects of CPAP and HFNO on the laryngeal and pharyngeal chemo- and mechanoreceptors, with subsequent hypoesthesia, delayed swallow reflex, weak cough, and breathing swallowing incoordination. In addition, Borders et al. [38] denoted that prolonged intubation may reduce laryngeal sensation, resulting in silent aspiration. Our results revealed that every 1-point increase in EAT-10 score increased the risk of aspiration by 1.31. This finding is in harmony with the Cheney et al. [39] study, which showed that aspiration was 2.2 times more common in dysphagic patients with an EAT-10 score greater than 15.

In post-COVID-19 patients, it appears crucial to assess swallowing function by screening tools as part of routine care, ideally early on, before starting oral intake, to prevent complications associated with OD. Because of the high rate of pooling of secretions in the hypopharynx, silent aspiration, and residues, FEES is recommended in patients who fail OD screening.

## Limitations

Findings must be interpreted in the context of the following limitations: First, there is a lack of longitudinal follow-up for the recovery pattern of dysphagia in the same group of patients across time. Evaluating the same patients at several intervals and more extended follow-up periods would provide more information regarding dysphagia progression and recovery. Second, considering the multifactorial nature of dysphagia in our patients, it was not possible to separate the effect of COVID-19 disease on swallowing function. The adjusted odds ratio was estimated in order to account for the confounding factors. Third, there is a lack of information about the dysphagia characteristics, evolution, and recovery in the dropout cases as well as in the group of patients who failed the screening after discharge and had a positive PCR, then later passed the screening after their PCR turned negative. We acknowledge the lack of our missing data handling before the initial analysis to address the potential bias introduced by dropouts, which could impact our findings. Fourth, compensatory therapies may not directly alter the anatomical or physiological features observed during FEES but can improve swallowing safety and efficiency over time. Such improvement could mask underlying physiological deficits that were more apparent in the absence of therapy. As stated by Newman et al. [40], increasing bolus viscosity impacts the physiology with increased lingual pressure patterns and controversial effects on hyoid displacements, oral and pharyngeal transit time, the onset of upper esophageal sphincter opening, and bolus velocity. However, we thought that implementing the compensatory therapies for a relatively short period (3–4 weeks) would not create lasting functional change.

## Conclusion

Swallowing assessment of the post-COVID-19 patients using FEES revealed several swallowing abnormalities at both the initial post-discharge evaluation and the subsequent follow-up evaluation. The combination of the following risk factors, “higher EAT-10 scores and higher respiratory rate,” predicted aspiration in post-COVID-19 patients by an overall percentage of 87.1%.

## Data Availability

Available (The datasets used and/or analysed during the current study are available from the corresponding author upon reasonable request).
